# Performance of the Mammoth Balloon Catheter in Patients with Severe Aortic Valve Stenosis Undergoing Percutaneous Treatment

**DOI:** 10.3390/jcm13195986

**Published:** 2024-10-08

**Authors:** Silvia Moscardelli, Rodolfo Caminiti, Carolina Montonati, Fabrizio Ceresa, Giuseppe De Blasio, Giampiero Vizzari, Dario Pellegrini, Mariano Pellicano, Giulio Guagliumi, Francesco Patanè, Maurizio Tespili, Antonio Micari, Alfonso Ielasi

**Affiliations:** 1 U.O. Cardiologia Ospedaliera, IRCCS Ospedale Galeazzi Sant’Ambrogio, 20157 Milan, Italy; silvia@moscardelli.it (S.M.); rodolfocaminiti@msn.com (R.C.); carolina.montonati@grupposandonato.it (C.M.); giuseppe.deblasio@grupposandonato.it (G.D.B.); dario.pellegrini@grupposandonato.it (D.P.); mariano.pellicano@grupposandonato.it (M.P.); giulio.guagliumi@grupposandonato.it (G.G.); tespili@katamail.com (M.T.); 2 Divisione di Cardiologia, Policlinico Madonna della Consolazione, 89124 Reggio Calabria, Italy; 3 Department of Cardiothoracic Surgery, Papardo Hospital, 98158 Messina, Italy; ceresa77@hotmail.com (F.C.); francescopatane@aopapardo.it (F.P.); 4 Interventional Cardiology Unit, Department of Clinical and Experimental Medicine, University of Messina, 98122 Messina, Italy; giampiero.vizzari@polime.it (G.V.); antonio.micari@unime.it (A.M.)

**Keywords:** aortic valve stenosis, trans-catheter aortic valve replacement, balloon aortic valvuloplasty, balloon catheter

## Abstract

**Background**: Balloon aortic valvuloplasty (BAV) is currently used as pre-treatment for patients undergoing trans-catheter aortic valve replacement (TAVR) as well as a stand-alone option for subjects with significant contraindications to TAVR. Mammoth is a newly available non-compliant balloon catheter (BC) included in the balloon-expandable Myval THV system (Meril Life Sciences Pvt. Ltd., India). As limited data on the performance of this BC are available, we here report the results following its use for BAV as pre-dilatation during TAVR or as a stand-alone procedure. **Methods**: A retrospective, single-center cohort analysis was performed on patients with severe aortic valve stenosis (AS) treated with the Mammoth BC at IRCCS Ospedale Galeazzi Sant’Ambrogio, Milan, Italy. The primary endpoint was technical success defined as successful Mammoth BC advancement across the AS followed by its full and homogeneous inflation without major complications such as aortic root/left ventricular outflow tract injury and/or stroke. **Results**: A total of 121 patients were treated by BAV with Mammoth BC during the study period. Among these, 105 patients underwent BAV pre-dilatation before TAVR while 16 patients underwent a stand-alone BAV procedure. Mammoth BC was delivered and successfully inflated at the target site in all of the 121 cases without BC-related complications (100% technical success). However, in the BAV “stand-alone group”, three patients required two different balloon sizes while in nine patients multiple rounds (two to three) of balloon inflation were needed to significantly lower the transvalvular gradient. No cases of aortic root injury or massive aortic regurgitation due to Mammoth BC-related aortic leaflet injury were reported while one major stroke occurred late after TAVR. No intra-procedural deaths occurred nor bleeding (BARC 3-4) or major vascular complication. **Conclusions**: Mammoth BC use in patients with severe AS proved safe and effective, either before TAVR or as a stand-alone procedure, expanding the range of available tools for structural operators.

## 1. Introduction

Transcatheter aortic valve replacement (TAVR) has become a widely accepted treatment option over surgical replacement for patients with severe, symptomatic aortic stenosis (AS), independently, by the surgical risk [1]. It has traditionally been a complex multistep procedure associated with certain challenges including the need for the implantation a new permanent pacemaker (PPM), significant paravalvular leak (PVL) risk, the risk of trans-catheter heart valve (THV) dislocation/migration, annular rupture, and the need for a second THV [2,3,4,5,6,7].

In an attempt to simplify the procedure, direct THV implantation without pre-dilatation balloon aortic valvuloplasty (BAV) is being favored as an attractive strategy for many TAVR procedures [7]. Nevertheless, there might be technical difficulties associated with direct THV deployment in heavily calcified and stenosed aortic valves, such as hemodynamic instability during device positioning, severe under-expansion or THV frame infolding, and inability to cross the native AS [8,9,10]. Hence, it might be helpful to plan TAVR with or without BAV based on the clinical (e.g., very high trans-valvular gradient or low left ventricle ejection fraction) and anatomical (e.g., bicuspid aortic valve, heavily calcified leaflets, or calcium protruding into the left ventricle outflow tract) characteristics of the patient observed on pre-procedural evaluation [7].

Moreover, there might be patients with severe AS who are unfit for TAVR due to severe comorbidities limiting short-to-mid-term life expectancy. In such patients, BAV can be performed as a stand-alone palliative procedure or as a bridge to final therapy. It allows these patients to undergo an urgent non-cardiac surgery if needed, with good immediate hemodynamic results [11]. BAV was introduced in 1986, and early reports showed poor outcomes. However, since then, there have been significant advances in BAV technologies (e.g., newer balloon catheter) and techniques, which have led to better results [12]. 

Mammoth is an over-the-wire balloon catheter (BC) (Meril Life Sciences Pvt. Ltd., Vapi, India) included in the balloon-expandable Myval THV system [13]. This tool can be used for BAV pre- or post-dilatation after TAVR or as a stand-alone procedure in extremely diseased patients. As limited data on the performance of this BC are actually available, we here present the outcomes of BAV as a stand-alone procedure or before/after TAVR using this novel interventional tool. 

## 2. Materials and Methods

### 2.1. Objective and Type of Study/Study Design

This was a retrospective, single-center cohort analysis conducted on patients with severe, native AS who underwent BAV as a stand-alone procedure or TAVR with pre-dilatation at IRCCS Ospedale Galeazzi Sant’Ambrogio, Milan, Italy, between September 2019 and March 2023. Only patients treated with the Mammoth balloon catheter (BC), available in the European market since September 2019, were included in this analysis. The choice of the Mammoth BC versus a different one available in the catheterization laboratory was left to the operator’s discretion. However, the Mammoth BC was used in all TAVR cases performed with pre-dilatation with Myval or Myval Octacor THV (Meril Life Sciences Pvt. Ltd., Vapi, India). 

The data collection complied with the Declaration of Helsinki and was approved by the local ethical committee and all patients provided written informed consent for the procedure and subsequent data collection based on local practice and/or local institutional review board approval. 

### 2.2. Study Device

**Figure 1:**
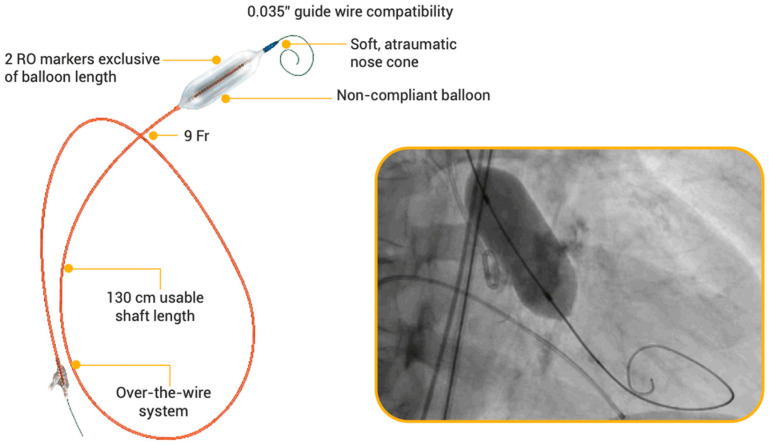
Mammoth over-the-wire balloon catheter system. RO: radiopaque.

Mammoth is a low-profile (9 French compatible) non-compliant, single-layer, over-the-wire BC with a usable shaft of 130 cm (Figure 1). 

**Figure 2:**
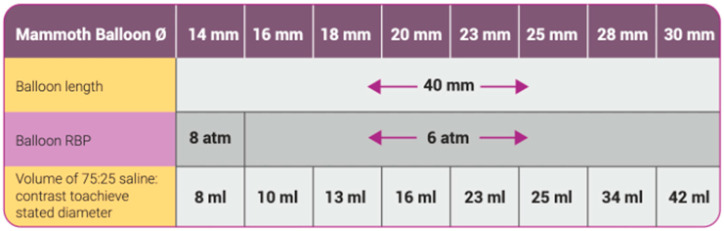
Mammoth balloon catheter system sizes.

Its composition material is Vestamid Care ML21; it is available in 6 different diameters (from 14 mm to 30 mm) with a length of 40 mm (Figure 2).

This BC has a soft, atraumatic tip and it is compatible with a 0.035″ guidewire having a minimum length of 260 cm. It has a specific indeflator depending on its diameter: up to 25 mm, the balloon inflator has a volume of 30 mL, and over 25 mm the balloon inflator has a volume of 60 mL.

The Mammoth BC has a double-lumen body with a balloon fixed in the distal part. The external lumen is used for filling and emptying the balloon. The internal catheter’s lumen enables the passage of the guidewire, which makes it possible to direct the catheter. Two X-ray-proof markers are helpful in properly positioning the balloon during the procedure.

Mammoth BC size was chosen for pre-dilatation or BAV alone according to the minimum aortic annulus diameter, the degree of leaflet calcification, and the nature (bicuspid or tricuspid) of the native aortic valve obtained at the pre-procedural multi-slice computed tomography (MSCT) for patients planned for TAVR, while trans-thoracic (TT) echocardiographic (and MSCT, whenever possible, according to the renal function of the patient) assessment for patients undergoing BAV as a stand-alone procedure. The balloon size for post-dilatation in patients undergoing TAVR was chosen according to the average diameter perimeter derived by MSCT. Aortic leaflet, annular, and left ventricular outflow tract calcifications were classified and graded by MSCT using a semiquantitative scoring system. Aortic valve calcification was graded semi-quantitatively as follows: grade 1, no calcification; grade 2, mildly calcified (small, isolated spots); grade 3, moderately calcified (multiple larger spots); and grade 4, heavily calcified (extensive calcifications of all cusps) [14,15].

Mammoth BC inflation was performed during rapid pacing of the right (lead in place) or left (wire) ventricle, usually at 180 to 200 bpm. The femoral puncture site was closed with a double Proglide (Abbott Vascular Abbott Vascular, Santa Clara, CA, USA) in case of TAVR or 8F Angioseal (Terumo Interventional Systems, Somerset, NJ, USA) in case of BAV only.

The primary outcome of the study was technical success defined as successful Mammoth BC advancement across the AS followed by its full and homogeneous inflation without major complications such as aortic root/left ventricle outflow tract injury and/or stroke.

Secondary outcomes were procedural success; a composite of technical success and trans-aortic valve pressure gradient reduction of at least 20 mmHg by TT echo or invasive hemodynamics; and free from death related to Mammoth BC usage. 

### 2.3. Statistical Analysis

The normality of the variables was assessed with the Shapiro–Wilk test and normal continuous variables were presented as mean ± standard deviation (SD). Categorical variables were expressed as numbers and percentages. All patients undergoing BAV pre-TAVR as a stand-alone procedure were included in the analysis.

Data were analyzed using SPSS version 26 (IBM, Armonk, NY, USA) and a general-purpose statistical software package called STATA (version: STATA 15.1, STATA Corp, College Station, TX, USA).

## 3. Results

Between September 2019 and March 2023, a total of 121 patients with severe, symptomatic AS underwent treatment with the Mammoth BC. The baseline clinical and imaging characteristics of the population are reported in Table 1.

Among these patients, BAV with Mammoth before TAVR was performed in 105 patients while 16 patients underwent a stand-alone BAV procedure. 

The mean age of the study population was 81.3 ± 8.9 years, and 66.5% were men. The mean Society of Thoracic Surgeons 30-day Predicted Risk of Mortality (STS PROM) score was 3.9 ± 2.1%. The baseline trans-thoracic (TT) echo mean aortic valve pressure gradient was 47.9 ± 17.5 mmHg and the mean aortic valve area (AVA) was 0.65 ± 0.20 cm^2^. 

The procedural characteristics of the population are reported in Table 2.

In all patients, the procedure was performed through femoral access under local anesthesia and conscious sedation. 

The Mammoth BC sizes used were 14 mm (n = 1, 0.8%), 16 mm (n = 5, 4.1%), 18 mm (n = 16, 13.2%), 20 mm (n = 23, 19%), 23 mm (n = 71, 58.6%), and 25 mm (n = 5, 4.1%). 

The Mammoth BC was delivered and successfully inflated at the target site across the stenosed aortic valve in all 121 cases without BC-related complications (100% technical success). 

However, in the BAV-alone sub-group, three patients required two different balloon sizes while in nine patients multiple rounds (two to three) of balloon inflation were needed to significantly lower the transvalvular gradient. 

In the 16 patients treated with BAV as a stand-alone procedure, the final post-procedure TT echocardiographic evaluation revealed a decrease in the mean pressure gradient of 23.1 ± 7.8 mmHg, and an increase in the aortic valve area of 0.3 ± 0.2 cm^2^ (Table 3).

In the overall population, no cases of aortic root injury or massive aortic regurgitation due to Mammoth BC-related aortic leaflet injury were reported, while one major stroke occurred (“late” after THV implantation and not directly related to Mammoth BC usage but related to the TAVR procedure itself). No intra-procedural deaths occurred nor bleeding BARC 3-4 or major vascular complication. Based on the reported results, procedural success was achieved in 111 of 121 treated patients (procedural success in the overall population: 91.7%; BAV “alone” group 93.8% vs. 91.9% BAV-TAVR group).

## 4. Discussion

Our study represents the largest report on patients with AS treated with the Mammoth BC so far. 

Although semi-compliant BCs are more often used for BAV as they apparently reduce structural injuries [16], we have shown that Mammoth non-compliant BC inflation either before TAVR or as a stand-alone procedure in extremely diseased patients is associated with good procedural results and a very low rate of complications. Although this is not a head-to-head comparison study between different balloons, the outcomes reported in our study are similar to those of semi-compliant balloons already available on the market [17].

According to their intrinsic features, non-compliant BCs expand uniformly over their longitudinal axis and generally cannot be expanded beyond a predetermined maximum diameter. Thus, they offer the advantage of deforming in a more predictable and stable manner during inflation than their semi-compliant competitors. Moreover, the main advantage of non-compliant balloons is their capability to target and treat highly calcified areas precisely, without affecting the surrounding healthy tissues. However, on the other hand, data coming from a single study comparing different semi- versus a single non-compliant (True, Bard Peripheral Vascular Inc., Tempe, AZ, USA) BC for pre-dilatation before the implantation of self-expandable THVs showed an increased occurrence of aortic rupture and higher rates of post-dilatation and conversion to open surgery in the non-compliant BC group [16] (Table 4).

Until now, the available, specific data on the clinical performance of the Mammoth BC were limited to case reports or small series (Table 5) while many studies reported the performance of the Myval BE THV without clearly stating whether the Mammooth BC was the only balloon used in the TAVR procedure [18,19,20,21,22].

### 4.1. Pre-TAVR BAV vs. Direct TAVR: Available Data and Current Recommendations

**Figure 3:**
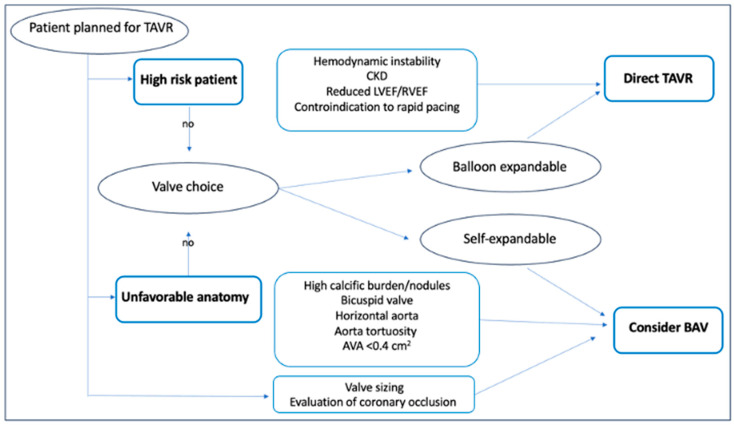
Proposed algorithm to identify patients who should be considered for BAV pre-dilatation or for direct TAVR.

Pre-dilatation BAV might be very helpful in cases where a self-expanding THV is planned to be implanted, prosthesis sizing is not completely established, or the potential risk of coronary obstruction requires further assessment [28]. Several observational studies have reported high success rates with direct TAVR compared to BAV before TAVR. The direct approach was associated with a lower procedural time, lower contrast volume injection, a lower rate of contrast-induced acute kidney injury (AKI), and a lower risk of PPM [29,30,31,32,33,34,35]. However, most of these were retrospective studies, with no prespecified criteria indicating preliminary BAV vs. direct TAVR; this aspect may have increased the risk of selection bias. A study that compared systematic pre-dilatation, selective pre-dilatation, and direct TAVR, reported similar procedural success rate, 30-day and 6-month mortality rates in all groups [32]. However, in patients with heavily calcified AS, selective pre-dilatation reduced the risk of THV malposition and need for a second THV [36]. In patients meeting all the following criteria: AVA > 0.4 cm^2^, central orifice, absence of severe calcification, mobility of aortic cusps not severely restricted, no left ventricular outflow tract calcification, absence of calcium nodules in the landing zone, and absence of severe aortic regurgitation, direct TAVR showed a high rate of success and low rates of post-dilatation, PVL, and PPM [37]. Islas et al. suggested similar echocardiography criteria to help identify patients for whom direct TAVR is unfavorable. These included: AVA < 0.4 cm^2^, irregular valve orifice, presence of calcium nodules, and leaflet calcification greater than grade 2 [38]. Based on these data, cases with severe or asymmetric aortic valve calcification, small AVA (<0.5 cm^2^), horizontal aorta or bicuspid valves should be considered for BAV [1,36]. The 2017 American College of Cardiology consensus document for the use of TAVR in the management of severe AS has also suggested that pre-dilation may be useful if the coronary ostia are low-lying, to assess the risk of coronary obstruction with THV implantation [39]. To summarize the available data and recommendations, McInerney et al. proposed an algorithm to identify patients who should be considered for BAV or for direct TAVR (Figure 3) [40].

### 4.2. BAV as Stand-Alone Procedure for Aortic Stenosis

Our study showed that, among patients who underwent standalone BAV, 18.7% required two different balloon sizes while in 56.2% of patients, multiple rounds (two to three) of balloon inflation were required to finally lower the transvalvular gradient. A decrease in the mean pressure gradient of 23.1 ± 7.8 mmHg, and an increase in the aortic valve area of 0.3 ± 0.2 cm^2^ was achieved. Kleczynski et al. [11] performed BAV mainly with VACS BC (Osypka Medical Inc. Berlin, Germarereny) as a rescue or bridge therapy in 374 patients reporting an intra-procedural mortality of 2.4%, and an in-hospital mortality of 5%, with 3.5% of patients requiring urgent cardiac surgery. Balloon rupture was reported in 6.1% of cases. In a more recent study, Bruno et al. [41] reported data on BAV alone in 174 patients who were unfit for TAVR. All procedures were performed with the Cristal BC (Balt), a semi-compliant balloon, mainly in its 20 mm diameter version. Invasive maximum and mean gradients were reduced from 56 ± 24 mmHg and 39 ± 17 mmHg to 28 ± 15 mmHg and 20 ± 12 mmHg, respectively. In-hospital mortality was 5%. A pooled analysis of >14,300 patients from studies on BAV published between 1991 and 2022, reported an intra-procedural mortality and an in-hospital mortality rates of 1.9% and 6.0% indicating that the procedure is safe. The BCs used included NuMED NuCLEUS, Cristal, Tyshak, and Zmed II. The decrease in the transvalvular pressure gradient has been shown to persist for at least 30 days after the procedure and is associated with considerable improvements in symptoms related to heart failure [12]. Despite studies showing the procedure to be safe, current American College of Cardiology/American Heart Association and ESC guidelines recommend (class II B) BAV only in patients with severe AS requiring urgent non-cardiac surgery [39,42]. Based on current evidence, BAV could be considered as a bridge-to-decision in high-risk patients with severe AS who cannot undergo TAVR immediately.

The present study has certain limitations related to its retrospective nature, the single-center experience and the BC selection (e.g., Mammoth versus others) being left to the operator’s discretion. Thus, there is a potential risk for selection bias, although the baseline characteristics of our patients highlight a high-risk cohort, in which BAV still achieved good results.

## 5. Conclusions

Our study shows that BAV alone or before TAVR using the Mammoth non-compliant BC is effective and safe in a real-world setting. A head-to-head comparison versus different semi-compliant/non-compliant BCs used for BAV is necessary to assess the potential advantages of this device over the others that are commercially available.

## Figures and Tables

**Table 1:** Baseline clinical and imaging characteristics of the population.

Baseline Clinical Characteristics	n = 121
Age (years), mean ± SD	81.3 ± 8.9
Male gender, n (%)	80 (66)
CAD, n (%)	72 (60)
Prior MI, n (%)	17 (14)
Prior PCI, n (%)	52 (43)
Prior CABG, n (%)	6 (5)
History of atrial fibrillation, n (%)	38 (31)
Prior PM/ICD, n (%)	6 (5)
COPD, n (%)	11 (9)
Peripheral artery disease, n (%)	31 (25.6)
Severe chronic kidney disease (eGFR ≤ 30 mL/min)	20 (16.5)
Severe chronic kidney disease (eGFR ≤ 30 mL/min) in BAV-only patients	10/16 (62.5)
**Cardiovascular risk factors**	
Diabetes mellitus, n (%)	25 (21)
Arterial hypertension, n (%)	109 (90)
Hypercholesterolemia, n (%)	73 (60)
Active or former smoker, n (%)	38 (31)
Severe obesity (BMI > 40 kg/m^2^) n (%)	2 (2)
NYHA class ≥ 3, n (%)	60 (50.5)
EuroSCORE II (%), mean ± SD	4.4 ± 1.9
STS-PROM score (%), mean ± SD	3.9 ± 2.1
**Baseline trans-thoracic echocardiography assessment**	**n = 121**
Trans-valvular gradient (mmHg), mean ± SD	47.9 ± 17.5
Aortic valve area (cm^2^), mean ± SD	0.65 ± 0.20
LVEF (%), mean ± SD	52.7 ± 14.8
Aortic regurgitation ≥ moderate, n (%)	29 (24)
**Baseline MSCT assessment**	**n = 111**
Aortic annulus diameter (mm), mean ± SD	23.6 ± 2.4
Aortic annulus area (mm^2^), mean ± SD	466.1 ± 106.5
Aortic annulus average perimeter (mm), mean ± SD	74.1 ± 6.8
Degree of moderate aortic leaflet calcification, n (%)	50 /111(45.0)
Degree of severe aortic leaflet calcification, n (%)	61/111 (54.9)
Degree of LVOT calcification ≥ moderate, n (%)	5/111 (4.5)
Left common femoral artery diameter, (mm) mean ± SD	6.4 ± 0.6
Right common femoral artery diameter, (mm) mean ± SD	6.8 ± 0.9

Abbreviations: SD: standard deviation; CAD: coronary artery disease, MI: myocardial infarction, PCI: percutaneous coronary intervention, CABG: coronary artery bypass graft, PM/ICD: pacemaker/implantable cardioverter–defibrillator, COPD: chronic obstructive pulmonary disease, SD: standard deviation, LVEF: left ventricular ejection fraction, MSCT: multi-slice computed tomography.

**Table 2:** The procedural characteristics of the population.

Procedural Characteristics	n = 121
Trans-femoral access, n (%)	121 (100)
BAV procedure time (mins), mean ± SD	19.4 ± 9.2
BAV fluoroscopy time (mins), mean ± SD	24.4 ± 12.8
BAV total contrast volume (ml), mean ± SD	24.3 ± 11.9
Local anesthesia plus mild sedation, n (%)	121 (100)
Safari S wire in the left ventricle, n (%)	91 (75.2)
Safari XS wire in the left ventricle, n (%)	23 (19)
Innowi wire in the left ventricle, n (%)	2 (1.6)
Lunderquist wire in the left ventricle, n (%)	5 (4.1)
Post-dilatation required after TAVR, n (%)	3/111 (2.7)

**Table 3:** Haemodynamic and echocardiographic assessment after BAV.

Haemodynamics	Overall Population n= 121
Baseline peak-to-peak transvalvular gradient (mmHg), mean ± SD	59.8 ± 22.6
Post-BAV peak-to-peak transvalvular gradient (mmHg), mean ± SD	24.3 ± 11.4
**Haemodynamics**	**BAV alone** **n = 16**
Baseline transvalvular gradient (mmHg), mean ± SD	42.9 ± 10.7
Post-BAV transvalvular gradient (mmHg), mean ± SD	21.0 ± 6.9
**Trans-thoracic Echocardiogram**	**BAV alone** **n = 16**
Baseline transvalvular gradient (mmHg), mean ± SD	44.1 ± 9.8
Post-procedural transvalvular gradient (mmHg), mean ± SD	21.0 ± 2.0
Baseline effective orifice area (mm^2^), mean ± SD	0.82 ± 0.20
Post-procedural effective orifice area (mm^2^)	1.12 ± 0.22

Abbreviations: SD, standard deviation.

**Table 4:** Different Type of Balloon Catheter Used for TAVR or BAV as Standalone Procedure.

Balloon Type	Balloon Name	Manufacturer	Balloon Length (cm) n=	Shaft Length (cm) n=	Rated Burst Pressure (atm)	Total Sizes n=	Material
Non- compliant	VACS III	Osypka Medical	2–6	100	4–15	18	NA
	Mammoth	Meril	4	130	24	6	Vestamid Care ML21
	Tyshak II	B. Braun Interventional Systems, Inc. (Bethlehem, PA, USA)	2–8	70–100	1.5–6	74	Polymeric, DEHP-free
	True Dilatation	Bard	4.5	110	6	9	Fiber
	Atlas Gold	Bard	2–4–6	80–120	16	40	NA
	Z-MED™	B. Braun Interventional Systems, Inc.	2–6	100	1.5–3	52	NA
	Z-MED™II				3–4	77	
	SIM-valve force	Simeks Medical	2–6	100	4, 6, 8, 12	65	Nylon 12
	NuCLEUS NuCLEUS X	B. Braun Interventional Systems, Inc.	3–6 4–6	110	2–9 2–4	54	NA
Semi- compliant	VACS II	Osypka Medical	2–6	100	1.5–6	17	NA
	Cristal	BALT extrusion	3–6	110	6	9	NA
	Valver	Balton	2.5–6	110	3–5	15	NA

**Table 5:** Available data (case reports or small series) on the clinical performance of the Mammoth balloon catheter.

Author and Year of Publication	Patient Details	Procedure	Outcomes
**Santos-Martınez et al., 2018** [23]	83-year-old male. NYHA class III due to severe AS. Mean gradient 78 mmHg and AVA 0.6 cm^2^ with preserved LVEF. Euroscore II 2.9%.	Pre-dilatation with an 18 mm Mammoth balloon catheter (BC), then a 24.5 mm Myval implanted	THV correctly positioned, with mild PVL. Mean gradient 9 mmHg. Discharged at dat 4 without complications
**Chopra et al., 2020** [24]	59-year-old male, post-renal transplant (on immunosuppressant therapy). NYHA Class III. Severe degenerative aortic valve disease, bicuspid leaflet with heavy calcification. Aortic valve mean gradient 70 mmHg and AVA 0.7 cm^2^ with normal LVEF. STS mortality score 4.8% with combined mortality and morbidity score 21.7%	Pre-dilatation with a 20 mm Mammoth BC, then a 23 mm Myval was deployed	Residual mean gradient of 4 mm Hg with no PVL
**Maluenda et al., 2020** [18]	14 patients with severe AS at high surgical risk. Mean age 82.5 ± 7.8 years. Mean STS score mortality 11.6 ± 5.1% at 30-day. Mean aortic valve gradient 47 ± 9 mm Hg. Mean AVA 0.6 ± 0.2 cm^2^. Mean aortic annulus area 435 ± 88 mm^2^. Mean aortic annular perimeter 71 ± 15 mm	Routine pre-dilatation with a Mammoth BC was recommended. Post-dilatation was recommended in cases with more than mild to moderate PVL	Device and procedural success 86%. Substantial drop in mean aortic gradient, persistent at 6-month follow-up without more than mild aortic regurgitation. Device failure in 2 patients, one due to delivery failure and the other due to ventricular embolization. One early death due to dissection/rupture of the aorta and 2 major hemorrhages.
**Gupta et al., 2020** [25]	75-year-old frail female with insulin-dependent diabetes mellitus and CKD. Severe AS with calcified left main distal and ostial left anterior descending artery lesions. NYHA Class. Maximum and mean gradient of 110 and 65 mmHg and AVA of 0.6 cm^2^. STS risk score of 16 and EUROSCORE risk of in-hospital mortality of 8.25%	Pre-dilatation with a 16 mm Mammoth BC followed by deployment of a 21 mm Myval THV	THV placed and expanded successfully without PVL. At 6-month follow-up, patient in NYHA Class I.
**Ray, 2020** [26]	71-year-old male presented in emergency with chest pain and severe shortness of breath. Severe calcific AS with peak gradient of 56 mm/Hg and mean gradient of 43 mm/Hg; severe systolic dysfunction	Pre-dilatation with a 16 mm Mammoth BC at 7 atm, then Myval 20 mm implantation	THV placed and expanded successfully. Mild PVL. Uneventful recovery.
**Arslan et al., 2021** [17]	83-year-old male, NYHA class III. Severe AS (mean gradient, 59 mm Hg; AVA: 0.8 cm^2^). Aortic annulus area and perimeter 508 mm^2^ and 81 mm, respectively. STS risk score 4.2. Left coronary ostium height, left leaflet length, sinus curvature length, and sinus of Valsalva diameter: 14.4 mm, 15.4 mm, 15.1 mm, and 34 mm, respectively. Bulky calcifications, more pronounced in the left aortic leaflet.	Pre-dilatation with 23 mm Mammoth BC.	No PVL, but partial eft main coronary artery (LMCA) obstruction. Despite repeated balloon inflations, LMCA recoil occurred. A 6.0×18 mm renal stent was successfully implanted in the LMCA. Uneventful recovery.
**De Toledo et al., 2022** [27]	73-year-old female. History of TAVR with a 25 mm Acurate Neo THV to treat severe symptomatic AS. Structural valve degeneration with severe aortic regurgitation (AR), intra and para-prosthetic. LVEF 74%.	Neo THV was crossed followed by a partially inflation of a 20 mm Mammoth BC to confirm central crossing. Myval 26 mm THV was implanted below the nadir of the leaflets of the degenerated THV with an oversizing to the annulus of 12.3%	Mean gradient of 3 mmHg, angiogram confirmed patency of coronary arteries, and no residual AR. At 1-month follow-up, the patient remained asymptomatic with mean gradient: 9 mmHg, AVA: 2.3 cm^2^, and no leaks.

## Data Availability

Not applicable.
